# Heyde‐Like Syndrome Manifesting as Massive Gastrointestinal Bleeding After TAVI in an Elderly Patient With Gastric Angiodysplasia

**DOI:** 10.1002/ccr3.72537

**Published:** 2026-04-09

**Authors:** Toshiki Setoguchi, Daisuke Fukamachi, Naoya Matsumoto, Yasuo Okumura

**Affiliations:** ^1^ Department of Cardiology Nihon University Hospital Tokyo Japan; ^2^ Division of Cardiology, Department of Medicine Nihon University Itabashi Hospital Tokyo Japan

**Keywords:** angiodysplasia, antiplatelet therapy, Heyde syndrome, TAVI

## Abstract

Gastrointestinal bleeding due to angiodysplasia in elderly patients with severe aortic stenosis may be overlooked outside of cardiology. Clinicians should remain vigilant, especially in TAVI candidates presenting with unexplained anemia, as early recognition can significantly impact management and outcomes.

An octogenarian woman was admitted for heart failure due to severe aortic stenosis (AS) and anemia. After stabilization and transfusions, upper endoscopy revealed angiodysplasia in the gastric mucosa (Figure 1A), raising suspicion of Heyde syndrome. No antithrombotic therapy had been used prior to admission.

**FIGURE 1:**
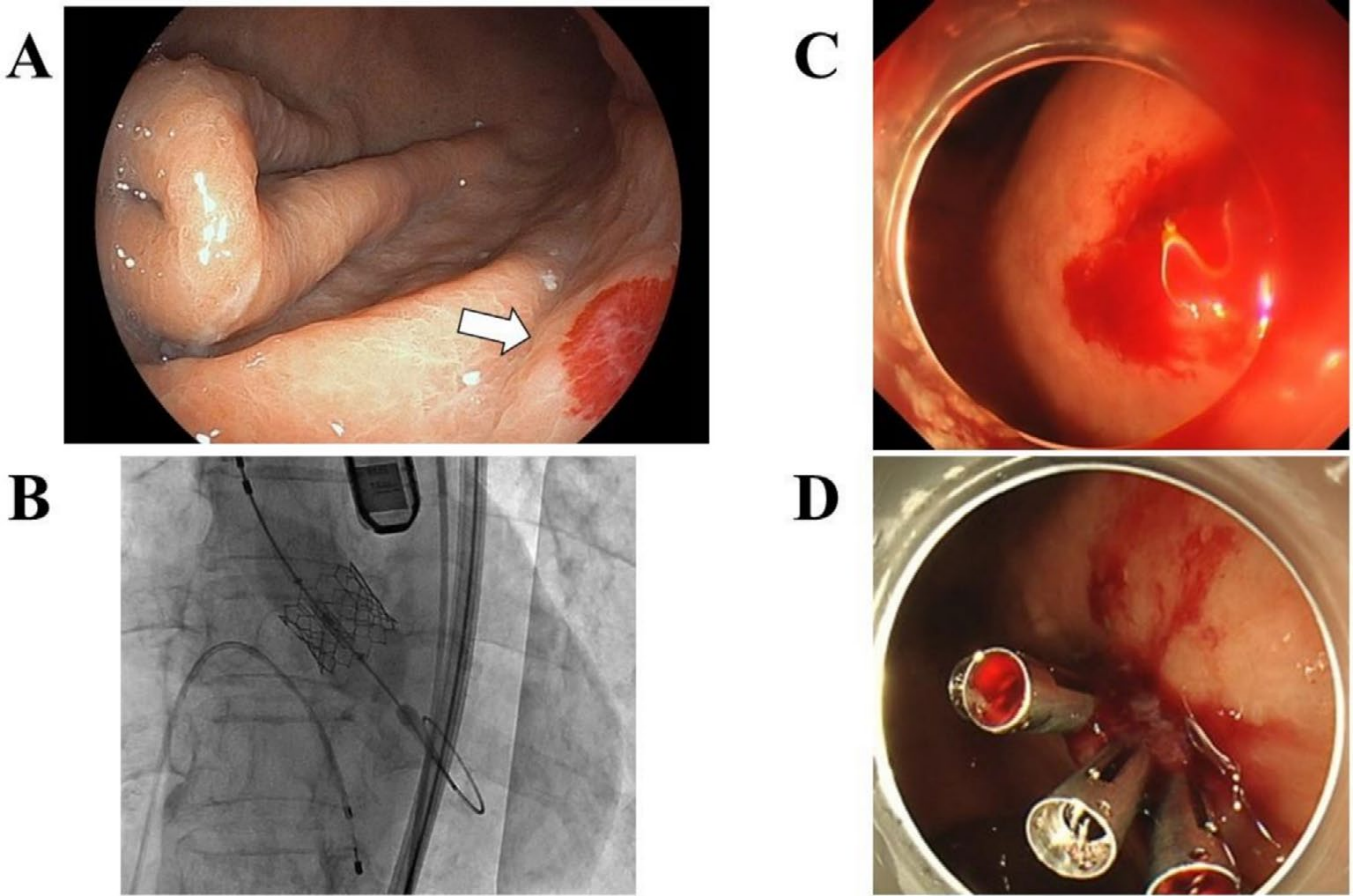
(A) Upper endoscopy performed during the evaluation of anemia revealed a vascular lesion consistent with angiodysplasia on the posterior wall of the gastric body, indicated by the arrow. (B) Transcatheter aortic valve implantation (TAVI) was performed to treat severe aortic stenosis. (C) Emergent upper endoscopy showed active bleeding from multiple gastric angiodysplasia lesions. (D) Hemostasis was successfully achieved using endoscopic clipping at the bleeding sites.

To address the AS, transcatheter aortic valve implantation (TAVI) was performed (Figure 1B). Aspirin and a proton pump inhibitor were started the following day. One week later, she developed massive hematemesis and hemorrhagic shock. Emergent endoscopy revealed active bleeding from multiple gastric angiodysplasias, which were managed with endoscopic clips (Figure 1C,D). She recovered and was discharged without recurrence of bleeding.

Heyde syndrome is classically defined by a triad of AS, gastrointestinal angiodysplasia, and acquired von Willebrand syndrome (AVWS) [1]. In our case, von Willebrand factor (vWF) testing was not performed prior to TAVI; a post‐bleeding vWF multimer analysis was normal. Thus, although the complete triad is unconfirmed, the clinical features are compatible with a Heyde‐like syndrome. Similar reports describe comparable presentations even in the absence of AVWS confirmation [2].

Importantly, recent data from a nationwide cohort of over 200,000 TAVI procedures indicate that gastrointestinal bleeding occurs in approximately 1% of patients, and angiodysplasia is a significant predictor of this complication [3]. This emphasizes the importance of careful antithrombotic management in elderly patients, especially those with known or suspected gastrointestinal vascular lesions.

## Author Contributions

**Toshiki Setoguchi:** data curation. **Daisuke Fukamachi:** conceptualization, project administration, writing – original draft. **Naoya Matsumoto:** writing – review and editing. **Yasuo Okumura:** writing – review and editing.

## Funding

The authors have nothing to report.

## Consent

Written informed consent was obtained from the patient to publish this report in accordance with the journal's patient consent policy.

## Conflicts of Interest

The authors declare no conflicts of interest.

## Data Availability

Data available on request from the authors.
